# 1,1′-(Diselanediylbis{[*P*,*P*-diphenyl-*N*-(tri­methyl­sil­yl)phospho­rimido­yl]methanylyl­idene})bis­[1,1-diphenyl-*N*-(tri­methyl­sil­yl)-λ^5^-phosphanamine] pentane disolvate

**DOI:** 10.1107/S1600536813032674

**Published:** 2013-12-07

**Authors:** Ramalingam Thirumoorthi, Tristram Chivers, Risto S. Laitinen

**Affiliations:** a Department of Chemistry, University of Calgary, 2500 University Drive NW, Calgary, Alberta T2N 1N4, Canada; b Department of Chemistry, PO Box 3000, University of Oulu, 90014 Oulu, Finland

## Abstract

The title compound, C_62_H_78_N_4_P_4_Se_2_Si_4_·2C_5_H_12_, is made up of two [SeC(PPh_2_NSiMe_3_)(PPh_2_NHSiMe_3_)] units related by an inversion center situated at the mid-point of the diselenide bond. It crystallized with two disordered mol­ecules of pentane used as solvent of crystallization. It is a rare example of an anti­periplanar diselenide and exhibits a long Se—Se bond of 2.4717 (8) Å. The Se—C bond length of 1.876 (5) Å is short in comparison with the range of values found for other diselenides (1.91–1.97 Å). The mol­ecule exhibits two intra­molecular N—H⋯N hydrogen bonds. In the crystal, there are no significant inter­molecular inter­actions present. One of the Me_3_Si– groups is disordered over two positions with a refined occupancy ratio of 0.708 (8):0.292 (8). The contribution of the disordered solvent to the scattering was removed with the SQUEEZE option of PLATON [Spek (2009 ▶). *Acta Cryst*. D**65**, 148–155]. The solvent contribution has been included in the reported mol­ecular weight and density.

## Related literature   

For the coordination chemistry of diselenides, see: Risto *et al.* (2011 ▶). For examples of anti­periplanar diselenides, see: Wagner *et al.* (1990 ▶); Dhau *et al.* (2011 ▶). For geometric parameters in organic diselenides, see: Dickson *et al.* (1999 ▶); Steudel *et al.* (1980 ▶); Schmidbaur *et al.* (1983 ▶); Konu *et al.* (2010 ▶); Back & Codding (1983 ▶); Pyykkö & Atsumi (2009 ▶). For the binding energies of organic dislenides, see; McDonough *et al.* (2005 ▶). For the synthesis of the reagent {Li_2_[C(PPh_2_NSiMe_3_)_2_]}, see: Kasani *et al.* (1999 ▶).
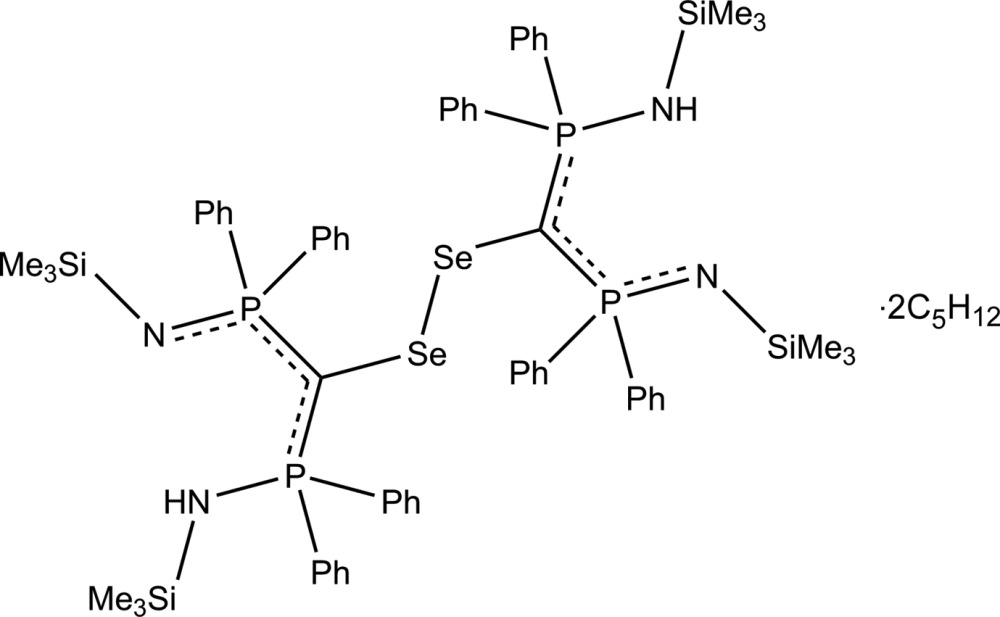


## Experimental   

### 

#### Crystal data  

C_62_H_78_N_4_P_4_Se_2_Si_4_·2C_5_H_12_
*M* *_r_* = 1417.74
Triclinic, 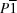

*a* = 10.2500 (3) Å
*b* = 13.4420 (3) Å
*c* = 14.6670 (4) Å
α = 65.611 (1)°
β = 85.525 (1)°
γ = 76.730 (2)°
*V* = 1791.03 (8) Å^3^
*Z* = 1
Mo *K*α radiation
μ = 1.23 mm^−1^
*T* = 173 K
0.22 × 0.15 × 0.09 mm

#### Data collection  

Nonius KappaCCD FR540C diffractometer
Absorption correction: multi-scan (*SORTAV*; Blessing, 1997 ▶) *T*_min_ = 0.773, *T*_max_ = 0.897
11811 measured reflections
6257 independent reflections
4902 reflections with *I* > 2σ(*I*)
*R*_int_ = 0.064

#### Refinement  

*R*[*F* ^2^ > 2σ(*F* ^2^)] = 0.068
*wR*(*F* ^2^) = 0.170
*S* = 1.04
6257 reflections
345 parameters
H atoms treated by a mixture of independent and constrained refinement
Δρ_max_ = 0.77 e Å^−3^
Δρ_min_ = −0.71 e Å^−3^

### 

Data collection: *COLLECT* (Nonius, 1999 ▶); cell refinement: *DENZO* (Otwinowski & Minor, 1997 ▶); data reduction: *SCALEPACK* (Otwinowski & Minor, 1997 ▶); program(s) used to solve structure: *SHELXS97* (Sheldrick, 2008 ▶); program(s) used to refine structure: *SHELXL97* (Sheldrick, 2008 ▶); molecular graphics: *DIAMOND* (Putz & Brandenburg, 2013 ▶); software used to prepare material for publication: *WinGX* (Farrugia, 2012 ▶).

## Supplementary Material

Crystal structure: contains datablock(s) global, I. DOI: 10.1107/S1600536813032674/su2665sup1.cif

Structure factors: contains datablock(s) I. DOI: 10.1107/S1600536813032674/su2665Isup2.hkl

Additional supporting information:  crystallographic information; 3D view; checkCIF report

## Figures and Tables

**Table 1:** Hydrogen-bond geometry (Å, °)

*D*—H⋯*A*	*D*—H	H⋯*A*	*D*⋯*A*	*D*—H⋯*A*
N2—H2*N*⋯N1	0.99 (8)	1.86 (8)	2.795 (8)	156 (6)

**Fig. 1.**
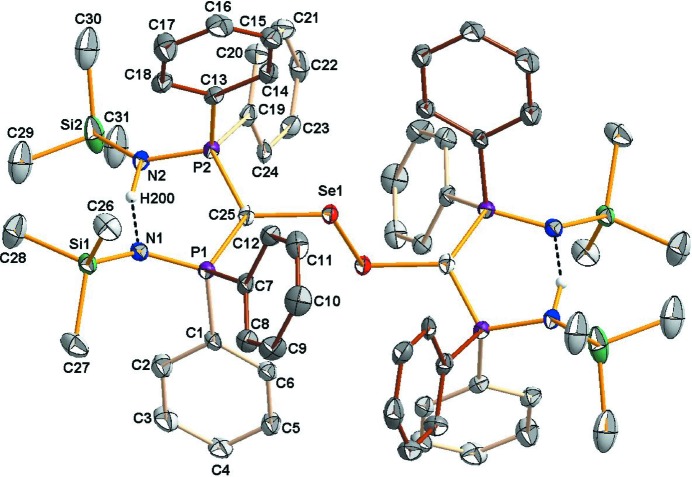
A view of the title molecule, with atom labelling. Displacement ellipsoids are drawn at the 50% probability level. The intramolecular N-H···N hydrogen bonds are shown as dashed lines (see Table 1 for details; only the major components of the disordered methyl groups on Si2 are shown; the C-bound H atoms have been omitted for clarity).

## supplementary crystallographic information

### 1. Comment

Organic diselenides are of interest from the viewpoint of coordination chemistry (Risto *et al.*, 2011) as well as for their structural aspects. The C—Se—Se—C torsion angle typically falls within a wide range of *ca.* 73–128° (Dickson *et al.*, 1999). The only examples of antiperiplanar diselenides (C—Se—Se—C = 180°) involve very bulky *R* groups, for example, (Me_3_Si)_3_CSeSeC(SiMe_3_)_3_ (*d*(Se—Se) = 2.388 (1) Å) (Wagner *et al.*, 1990), or intramolecular heteroatom coordination to the Se centers as in bis(3,5-dimethyl-2-pyridyl)diselenide (*d*(Se—Se) = 2.352 (2) Å) (Dhau *et al.*, 2011). In this work we unexpectedly isolated a small amount of the title diselenide as red crystals from the reaction of {Li_2_[C(PPh_2_NSiMe_3_)_2_]} with SeCl_4_ (1:1 molar ratio) in pentane.

The crystal structure analysis of the title compound revealed a centrosymmetric dimer in which a diselenido (–Se—Se-) unit bridges two monoprotonated units (Fig. 1). The torsion angle C25—Se—Se^i^—C25^i^ is 180 °, as a result of the inversion centre, and the two anionic ligands are in a *trans* orientation with respect to –Se—Se- bridge. The Se—Se bond length of 2.4717 (8) Å is substantially longer than those in typical diaryl diselenides (range 2.29–2.35 Å) [Dickson *et al.*, 1999]. At the same time the Se—C bond of 1.876 (5) Å is short in comparison with the range of values found for other diselenides (1.91–1.97 Å) (Back & Codding, 1983) and the calculated value of 1.91 Å (Pyykkö & Atsumi, 2009). This bonding arrangement is reminiscent of the alternation of the S—S bond lengths in cycloheptasulfur, which has been rationalized in terms of p lone pair repulsions of the neighbouring sulfur atoms due to the torsion angle of 0° and hyperconjugation (Steudel *et al.*, 1980). We also note that the Se—Se bond lengths of 2.492 (2) Å found for the dication [(Ph_3_P)_2_CSe-SeC(PPh_3_)_2_]^2+^ (Schmidbaur *et al.*, 1983) and 2.508 (1) Å exhibited by the dilithium complex {[Li(TMEDA)]_2_[(SPh_2_P)_2_CSe-SeC(PPh_2_S)_2_]} (Konu *et al.*, 2010) are comparable to that of the title compound. In the latter case, the Se—Se bonding interaction is due solely to the poor overlap of the SOMOs of the anion radicals. Consequently the calculated binding energy is small (90 kJ mol^-1^) compared to typical values for organic diselenides, *e.g.* 172 kJ mol^-1^ for PhSe-SePh (McDonough *et al.*, 2005). The Se—C distance of 1.885 (3) Å in [{Li(TMEDA)}_2_{(SPh_2_P)_2_CSe-SeC(PPh_2_S)_2_}] (Konu *et al.*, 2010) is also comparable with that in the title compound.

The protonation of one of the nitrogen atoms, N1, is evident from the disparity of *ca* 0.07 Å in the P—N bond lengths, and smaller differences in the Si—N and P—C(Se) distances of 0.038 Å and 0.036 Å, respectively. Intramolecular hydrogen bonding between the N—H functionality and two-coordinate nitrogen atom (1.981 (15) Å) is observed (Table 1). The geometry at the three-coordinate carbon atom is almost planar (Σ<C(25) = 356.1°).

In the crystal, there are no significant intermolecular interactions present.

### 2. Experimental

The reagent {Li_2_[C(PPh_2_NSiMe_3_)_2_]} (I), was prepared according to the literature procedure (Kasani *et al.*, 1999). A mixture of (I) (0.254 g, 0.445 mmol) and SeCl_4_ (0.098 g, 0.445 mmol) was placed in a 50 ml round-bottomed flask under an argon atmosphere using standard Schlenk techniques. Pentane (10 ml) was added drop-wise over a period of 15 min. at 195 K. The solution was then allowed to reach room temperature and stirred for a further 8 h after which it was filtered, to remove LiCl, and gave an orange solution. The ^31^P NMR spectrum of the filtrate (400 MHz, pentane, 298 K) revealed a complex mixture of products with resonances at δ 4.99 (*s*), 1.14(*s*), 3.87(*s*), 5.40 (d, J = 5.0 Hz), 7.83(d, J = 5.0 Hz), 10.23(*s*), and 18.51(*s*). The filtrate was allowed to evaporate slowly at room temperature under an argon atmosphere to produce a few red crystals of the title compound after 45 days. Further details of the synthetic procedure are available in the archived CIF.

### 3. Refinement

The hydrogen atom bound to atom N2 was located in the difference Fourier map and refined with U_iso_(H) = 0.05 Å^2^. All C-bound H atoms were placed in idealized positions and treated as riding atoms: C-H = 0.95 and 0.98 Å for CH and CH_3_ H atoms, respectively, with U_iso_(H) = 1.5U_eq_(C-methyl) and = 1.2U_eq_(C) for other H atoms. The Me_3_Si group involving Si2 is disordered with two alternative orientations, atoms C30/C31:C32/C33 with a refined occupany ratio of 0.708 (8):0.298 (8), and their anisotropic displacement parameters were made equal. The disordered solvent pentane molecules could not be refined satisfactorily and were excluded using the SQUEEZE routine in *PLATON* (Spek, 2009); solvent-accessible void space of 249 Å^3^ corresponding to 85 electron count/cell. This is equivalent to two pentane solvent molecules (84 electrons) per unit cell.

### Crystal data

**Table:**

C_62_H_78_N_4_P_4_Se_2_Si_4_·2C_5_H_12_	*Z* = 1
*M**_r_* = 1417.74	*F*(000) = 746
Triclinic, *P*1	*D*_x_ = 1.314 Mg m^−^^3^
Hall symbol: -P 1	Mo *K*α radiation, λ = 0.71073 Å
*a* = 10.2500 (3) Å	Cell parameters from 6257 reflections
*b* = 13.4420 (3) Å	θ = 1.7–25.0°
*c* = 14.6670 (4) Å	µ = 1.23 mm^−^^1^
α = 65.611 (1)°	*T* = 173 K
β = 85.525 (1)°	Block, red
γ = 76.730 (2)°	0.22 × 0.15 × 0.09 mm
*V* = 1791.03 (8) Å^3^	

### Data collection

**Table:**

Nonius KappaCCD FR540C diffractometer	4902 reflections with *I* > 2σ(*I*)
Horizonally mounted graphite crystal monochromator	*R*_int_ = 0.064
φ scans, and ω scans with κ offsets	θ_max_ = 25.0°, θ_min_ = 1.7°
Absorption correction: multi-scan (*SORTAV*; Blessing, 1997)	*h* = −12→12
*T*_min_ = 0.773, *T*_max_ = 0.897	*k* = −15→15
11811 measured reflections	*l* = −17→17
6257 independent reflections	

### Refinement

**Table:**

Refinement on *F*^2^	Primary atom site location: structure-invariant direct methods
Least-squares matrix: full	Secondary atom site location: difference Fourier map
*R*[*F*^2^ > 2σ(*F*^2^)] = 0.068	Hydrogen site location: inferred from neighbouring sites
*wR*(*F*^2^) = 0.170	H atoms treated by a mixture of independent and constrained refinement
*S* = 1.04	*w* = 1/[σ^2^(*F*_o_^2^) + (0.0591*P*)^2^ + 10.1653*P*] where *P* = (*F*_o_^2^ + 2*F*_c_^2^)/3
6257 reflections	(Δ/σ)_max_ < 0.001
345 parameters	Δρ_max_ = 0.77 e Å^−^^3^
0 restraints	Δρ_min_ = −0.71 e Å^−^^3^

### Special details

**Table:**

Experimental. Some details of the procedure for the synthesis of the title compound: Solvents were dried over and distilled from Na/benzophenone (toluene). t–BuLi (1.7 M in pentane), CH_2_(PPh_2_)_2_, and azidotrimethylsilane were purchased from Aldrich Chemical Co. and were used without further purification. ^31^P NMR spectra were recorded on Bruker 400 NMR spectrometers. ^31^P NMR chemical shifts were referenced externally to 85% H_3_PO_4_ (0 p.p.m.).
Geometry. Bond distances, angles etc. have been calculated using the rounded fractional coordinates. All su's are estimated from the variances of the (full) variance-covariance matrix. The cell esds are taken into account in the estimation of distances, angles and torsion angles
Refinement. Refinement of *F*^2^ against ALL reflections. The weighted *R*-factor *wR* and goodness of fit *S* are based on *F*^2^, conventional *R*-factors *R* are based on *F*, with *F* set to zero for negative *F*^2^. The threshold expression of *F*^2^ > σ(*F*^2^) is used only for calculating *R*-factors(gt) *etc*. and is not relevant to the choice of reflections for refinement. *R*-factors based on *F*^2^ are statistically about twice as large as those based on *F*, and *R*- factors based on ALL data will be even larger.

### Fractional atomic coordinates and isotropic or equivalent isotropic displacement parameters (Å^2^)

**Table:**

	*x*	*y*	*z*	*U*_iso_*/*U*_eq_	Occ. (<1)
Se1	0.62199 (5)	0.46193 (4)	0.01409 (4)	0.0187 (2)	
P1	0.66004 (14)	0.20765 (11)	0.15809 (10)	0.0169 (4)	
P2	0.68890 (14)	0.37513 (11)	0.23793 (10)	0.0176 (4)	
Si1	0.82048 (16)	−0.00542 (12)	0.31167 (12)	0.0221 (5)	
Si2	0.6027 (2)	0.28692 (16)	0.46105 (14)	0.0448 (7)	
N1	0.7173 (4)	0.1237 (4)	0.2656 (3)	0.0196 (12)	
N2	0.6632 (5)	0.2815 (4)	0.3492 (3)	0.0204 (14)	
C1	0.5008 (5)	0.1850 (4)	0.1340 (4)	0.0174 (16)	
C2	0.4386 (6)	0.1116 (5)	0.2129 (5)	0.0289 (17)	
C3	0.3148 (6)	0.0946 (5)	0.1980 (5)	0.036 (2)	
C4	0.2537 (6)	0.1490 (5)	0.1036 (5)	0.0300 (19)	
C5	0.3150 (6)	0.2206 (5)	0.0253 (4)	0.0260 (17)	
C6	0.4372 (5)	0.2381 (4)	0.0400 (4)	0.0213 (17)	
C7	0.7647 (5)	0.1909 (4)	0.0565 (4)	0.0202 (17)	
C8	0.7466 (6)	0.1186 (5)	0.0134 (4)	0.0250 (17)	
C9	0.8349 (6)	0.1011 (5)	−0.0570 (5)	0.031 (2)	
C10	0.9398 (6)	0.1555 (5)	−0.0885 (5)	0.0330 (19)	
C11	0.9581 (6)	0.2261 (5)	−0.0477 (5)	0.0297 (17)	
C12	0.8732 (6)	0.2440 (5)	0.0255 (4)	0.0246 (17)	
C13	0.8667 (5)	0.3776 (5)	0.2288 (4)	0.0205 (17)	
C14	0.9151 (6)	0.4578 (5)	0.1462 (4)	0.0257 (17)	
C15	1.0513 (6)	0.4514 (5)	0.1330 (5)	0.0289 (17)	
C16	1.1417 (6)	0.3631 (5)	0.2016 (5)	0.034 (2)	
C17	1.0965 (6)	0.2834 (5)	0.2835 (5)	0.0364 (19)	
C18	0.9581 (6)	0.2898 (5)	0.2983 (4)	0.0254 (17)	
C19	0.5998 (6)	0.5112 (4)	0.2292 (4)	0.0211 (17)	
C20	0.6627 (7)	0.5828 (5)	0.2472 (5)	0.031 (2)	
C21	0.5886 (7)	0.6841 (5)	0.2453 (5)	0.0321 (19)	
C22	0.4515 (7)	0.7124 (5)	0.2264 (5)	0.0372 (19)	
C23	0.3872 (7)	0.6421 (5)	0.2097 (4)	0.0305 (19)	
C24	0.4621 (6)	0.5401 (4)	0.2121 (4)	0.0234 (17)	
C25	0.6376 (5)	0.3468 (4)	0.1432 (4)	0.0189 (17)	
C26	0.9939 (6)	−0.0041 (5)	0.2624 (5)	0.0345 (19)	
C27	0.7579 (7)	−0.1112 (5)	0.2845 (5)	0.037 (2)	
C28	0.8339 (8)	−0.0567 (6)	0.4512 (5)	0.046 (3)	
C29	0.6230 (9)	0.1439 (7)	0.5524 (6)	0.061 (2)	
C30	0.7320 (13)	0.3449 (10)	0.5058 (8)	0.061 (2)	0.708 (8)
C31	0.4399 (13)	0.3696 (10)	0.4602 (8)	0.061 (2)	0.708 (8)
C32	0.581 (3)	0.409 (2)	0.4776 (19)	0.061 (2)	0.292 (8)
C33	0.394 (3)	0.287 (3)	0.4303 (19)	0.061 (2)	0.292 (8)
H2	0.48110	0.07320	0.27700	0.0350*	
H5	0.27280	0.25790	−0.03900	0.0310*	
H6	0.47900	0.28730	−0.01470	0.0250*	
H8	0.67370	0.08190	0.03300	0.0300*	
H9	0.82340	0.05080	−0.08440	0.0380*	
H10	0.99870	0.14390	−0.13820	0.0390*	
H3	0.27200	0.04580	0.25220	0.0430*	
H4	0.16950	0.13670	0.09320	0.0360*	
H14	0.85370	0.51740	0.09850	0.0310*	
H15	1.08310	0.50720	0.07720	0.0350*	
H16	1.23530	0.35780	0.19160	0.0410*	
H17	1.15890	0.22370	0.33030	0.0430*	
H18	0.92690	0.23490	0.35520	0.0300*	
H20	0.75650	0.56240	0.26080	0.0370*	
H21	0.63140	0.73360	0.25680	0.0390*	
H22	0.40100	0.78180	0.22480	0.0440*	
H23	0.29330	0.66250	0.19690	0.0360*	
H24	0.41850	0.49020	0.20180	0.0280*	
H26A	0.99130	0.01940	0.18970	0.0520*	
H26B	1.05060	−0.07940	0.29370	0.0520*	
H26C	1.03080	0.04830	0.27800	0.0520*	
H27A	0.66800	−0.11570	0.31160	0.0550*	
H27B	0.81870	−0.18460	0.31560	0.0550*	
H27C	0.75430	−0.08860	0.21190	0.0550*	
H28A	0.86960	−0.00440	0.46780	0.0700*	
H28B	0.89420	−0.13080	0.47860	0.0700*	
H28C	0.74490	−0.06180	0.48010	0.0700*	
H29A	0.71770	0.10600	0.55770	0.0920*	
H29B	0.56960	0.10490	0.53140	0.0920*	
H29C	0.59250	0.14320	0.61770	0.0920*	
H30A	0.81700	0.28940	0.52440	0.0920*	0.708 (8)
H30B	0.69690	0.36000	0.56400	0.0920*	0.708 (8)
H30C	0.74680	0.41430	0.45150	0.0920*	0.708 (8)
H31A	0.38760	0.32550	0.51430	0.0920*	0.708 (8)
H31B	0.39500	0.39310	0.39580	0.0920*	0.708 (8)
H31C	0.44750	0.43590	0.47010	0.0920*	0.708 (8)
H2N	0.667 (7)	0.215 (6)	0.335 (5)	0.0500*	
H11	1.03010	0.26360	−0.06960	0.0360*	
H12	0.88870	0.29190	0.05440	0.0290*	
H32A	0.50750	0.46490	0.43410	0.0920*	0.292 (8)
H32B	0.66390	0.43720	0.46040	0.0920*	0.292 (8)
H32C	0.56000	0.39470	0.54760	0.0920*	0.292 (8)
H33A	0.35630	0.23870	0.49180	0.0920*	0.292 (8)
H33B	0.39460	0.25770	0.37880	0.0920*	0.292 (8)
H33C	0.33990	0.36310	0.40660	0.0920*	0.292 (8)

### Atomic displacement parameters (Å^2^)

**Table:**

	*U*^11^	*U*^22^	*U*^33^	*U*^12^	*U*^13^	*U*^23^
Se1	0.0189 (3)	0.0179 (3)	0.0156 (3)	−0.0032 (2)	−0.0016 (2)	−0.0033 (2)
P1	0.0168 (7)	0.0161 (7)	0.0175 (7)	−0.0032 (6)	−0.0008 (6)	−0.0066 (6)
P2	0.0211 (7)	0.0178 (7)	0.0139 (7)	−0.0054 (6)	−0.0003 (6)	−0.0058 (6)
Si1	0.0204 (8)	0.0171 (8)	0.0240 (8)	−0.0023 (6)	−0.0017 (7)	−0.0043 (6)
Si2	0.0679 (15)	0.0298 (10)	0.0244 (10)	−0.0030 (10)	0.0196 (10)	−0.0063 (8)
N1	0.020 (2)	0.018 (2)	0.020 (2)	−0.0059 (19)	0.0012 (19)	−0.0063 (19)
N2	0.027 (3)	0.022 (2)	0.013 (2)	−0.009 (2)	0.0041 (19)	−0.0068 (19)
C1	0.015 (3)	0.012 (2)	0.022 (3)	0.000 (2)	−0.001 (2)	−0.005 (2)
C2	0.024 (3)	0.028 (3)	0.029 (3)	−0.005 (3)	−0.002 (3)	−0.006 (3)
C3	0.023 (3)	0.035 (4)	0.042 (4)	−0.013 (3)	0.001 (3)	−0.005 (3)
C4	0.018 (3)	0.028 (3)	0.040 (4)	−0.008 (3)	−0.007 (3)	−0.007 (3)
C5	0.020 (3)	0.025 (3)	0.026 (3)	−0.002 (2)	−0.005 (2)	−0.004 (3)
C6	0.020 (3)	0.020 (3)	0.023 (3)	−0.006 (2)	0.001 (2)	−0.007 (2)
C7	0.016 (3)	0.017 (3)	0.021 (3)	0.002 (2)	−0.006 (2)	−0.003 (2)
C8	0.022 (3)	0.023 (3)	0.029 (3)	−0.002 (2)	0.000 (3)	−0.011 (3)
C9	0.035 (4)	0.029 (3)	0.035 (4)	0.001 (3)	0.001 (3)	−0.022 (3)
C10	0.025 (3)	0.033 (3)	0.037 (4)	0.007 (3)	0.005 (3)	−0.018 (3)
C11	0.017 (3)	0.031 (3)	0.031 (3)	0.000 (3)	0.004 (3)	−0.006 (3)
C12	0.019 (3)	0.023 (3)	0.028 (3)	−0.002 (2)	−0.005 (2)	−0.007 (2)
C13	0.022 (3)	0.023 (3)	0.019 (3)	−0.006 (2)	−0.002 (2)	−0.010 (2)
C14	0.025 (3)	0.023 (3)	0.025 (3)	−0.005 (2)	−0.005 (3)	−0.005 (2)
C15	0.030 (3)	0.031 (3)	0.028 (3)	−0.014 (3)	0.003 (3)	−0.011 (3)
C16	0.025 (3)	0.045 (4)	0.037 (4)	−0.018 (3)	0.009 (3)	−0.018 (3)
C17	0.028 (3)	0.032 (3)	0.039 (4)	−0.001 (3)	−0.002 (3)	−0.007 (3)
C18	0.020 (3)	0.029 (3)	0.025 (3)	−0.005 (3)	−0.005 (2)	−0.008 (3)
C19	0.027 (3)	0.021 (3)	0.014 (3)	−0.001 (2)	0.000 (2)	−0.008 (2)
C20	0.038 (4)	0.028 (3)	0.031 (4)	−0.011 (3)	−0.001 (3)	−0.014 (3)
C21	0.043 (4)	0.031 (3)	0.030 (3)	−0.016 (3)	0.002 (3)	−0.016 (3)
C22	0.055 (4)	0.022 (3)	0.025 (3)	0.005 (3)	0.000 (3)	−0.007 (3)
C23	0.036 (4)	0.031 (3)	0.022 (3)	−0.001 (3)	−0.008 (3)	−0.010 (3)
C24	0.035 (3)	0.018 (3)	0.012 (3)	−0.004 (2)	0.000 (2)	−0.002 (2)
C25	0.018 (3)	0.018 (3)	0.016 (3)	−0.002 (2)	−0.002 (2)	−0.003 (2)
C26	0.020 (3)	0.031 (3)	0.048 (4)	−0.002 (3)	0.004 (3)	−0.014 (3)
C27	0.034 (4)	0.021 (3)	0.053 (4)	−0.004 (3)	0.000 (3)	−0.014 (3)
C28	0.051 (5)	0.041 (4)	0.034 (4)	0.010 (3)	−0.010 (3)	−0.011 (3)
C29	0.073 (4)	0.066 (4)	0.027 (3)	−0.004 (3)	0.011 (3)	−0.009 (3)
C30	0.073 (4)	0.066 (4)	0.027 (3)	−0.004 (3)	0.011 (3)	−0.009 (3)
C31	0.073 (4)	0.066 (4)	0.027 (3)	−0.004 (3)	0.011 (3)	−0.009 (3)
C32	0.073 (4)	0.066 (4)	0.027 (3)	−0.004 (3)	0.011 (3)	−0.009 (3)
C33	0.073 (4)	0.066 (4)	0.027 (3)	−0.004 (3)	0.011 (3)	−0.009 (3)

### Geometric parameters (Å, º)

**Table:**

Se1—C25	1.876 (5)	C2—H2	0.9500
Se1—Se1^i^	2.4717 (8)	C3—H3	0.9500
P1—N1	1.573 (4)	C4—H4	0.9500
P1—C1	1.814 (6)	C5—H5	0.9500
P1—C7	1.827 (6)	C6—H6	0.9500
P1—C25	1.753 (6)	C8—H8	0.9500
P2—N2	1.645 (5)	C9—H9	0.9500
P2—C13	1.824 (6)	C10—H10	0.9500
P2—C19	1.804 (6)	C11—H11	0.9500
P2—C25	1.731 (6)	C12—H12	0.9500
Si1—N1	1.699 (5)	C14—H14	0.9500
Si1—C26	1.867 (7)	C15—H15	0.9500
Si1—C27	1.876 (7)	C16—H16	0.9500
Si1—C28	1.876 (7)	C17—H17	0.9500
Si2—N2	1.732 (5)	C18—H18	0.9500
Si2—C29	1.808 (9)	C20—H20	0.9500
Si2—C30	1.956 (14)	C21—H21	0.9500
Si2—C31	1.778 (14)	C22—H22	0.9500
Si2—C32	1.72 (3)	C23—H23	0.9500
Si2—C33	2.22 (3)	C24—H24	0.9500
N2—H2N	0.99 (8)	C26—H26A	0.9800
C1—C2	1.396 (9)	C26—H26B	0.9800
C1—C6	1.396 (8)	C26—H26C	0.9800
C2—C3	1.389 (9)	C27—H27A	0.9800
C3—C4	1.390 (9)	C27—H27B	0.9800
C4—C5	1.376 (9)	C27—H27C	0.9800
C5—C6	1.375 (8)	C28—H28A	0.9800
C7—C12	1.404 (8)	C28—H28B	0.9800
C7—C8	1.410 (9)	C28—H28C	0.9800
C8—C9	1.378 (9)	C29—H29A	0.9800
C9—C10	1.382 (9)	C29—H29B	0.9800
C10—C11	1.365 (10)	C29—H29C	0.9800
C11—C12	1.393 (9)	C30—H30A	0.9800
C13—C14	1.396 (8)	C30—H30B	0.9800
C13—C18	1.399 (8)	C30—H30C	0.9800
C14—C15	1.382 (9)	C31—H31A	0.9800
C15—C16	1.391 (10)	C31—H31B	0.9800
C16—C17	1.372 (9)	C31—H31C	0.9800
C17—C18	1.407 (9)	C32—H32A	0.9800
C19—C24	1.389 (9)	C32—H32B	0.9800
C19—C20	1.390 (9)	C32—H32C	0.9800
C20—C21	1.389 (10)	C33—H33A	0.9800
C21—C22	1.387 (10)	C33—H33B	0.9800
C22—C23	1.375 (10)	C33—H33C	0.9800
C23—C24	1.397 (9)		
			
Se1^i^—Se1—C25	104.37 (16)	C7—C8—H8	120.00
N1—P1—C1	111.4 (2)	C9—C8—H8	120.00
N1—P1—C7	113.9 (3)	C8—C9—H9	119.00
N1—P1—C25	111.2 (3)	C10—C9—H9	119.00
C1—P1—C7	103.4 (3)	C9—C10—H10	120.00
C1—P1—C25	108.6 (3)	C11—C10—H10	120.00
C7—P1—C25	108.1 (3)	C10—C11—H11	119.00
N2—P2—C13	108.3 (3)	C12—C11—H11	119.00
N2—P2—C19	108.3 (3)	C7—C12—H12	120.00
N2—P2—C25	111.6 (3)	C11—C12—H12	120.00
C13—P2—C19	106.6 (3)	C13—C14—H14	120.00
C13—P2—C25	111.1 (3)	C15—C14—H14	120.00
C19—P2—C25	110.7 (3)	C14—C15—H15	120.00
N1—Si1—C26	112.5 (3)	C16—C15—H15	120.00
N1—Si1—C27	113.1 (3)	C15—C16—H16	120.00
N1—Si1—C28	108.7 (3)	C17—C16—H16	120.00
C26—Si1—C27	107.6 (3)	C16—C17—H17	120.00
C26—Si1—C28	107.1 (3)	C18—C17—H17	120.00
C27—Si1—C28	107.6 (3)	C13—C18—H18	120.00
N2—Si2—C29	106.8 (4)	C17—C18—H18	120.00
N2—Si2—C30	105.6 (4)	C19—C20—H20	120.00
N2—Si2—C31	118.1 (4)	C21—C20—H20	120.00
N2—Si2—C32	120.2 (9)	C20—C21—H21	120.00
N2—Si2—C33	94.3 (8)	C22—C21—H21	120.00
C29—Si2—C30	103.6 (5)	C21—C22—H22	119.00
C29—Si2—C31	113.1 (5)	C23—C22—H22	119.00
C29—Si2—C32	130.2 (9)	C22—C23—H23	120.00
C29—Si2—C33	89.7 (9)	C24—C23—H23	121.00
C30—Si2—C31	108.5 (6)	C19—C24—H24	120.00
C32—Si2—C33	101.7 (15)	C23—C24—H24	120.00
P1—N1—Si1	133.9 (3)	Si1—C26—H26A	109.00
P2—N2—Si2	134.8 (4)	Si1—C26—H26B	109.00
P2—N2—H2N	102 (4)	Si1—C26—H26C	109.00
Si2—N2—H2N	122 (4)	H26A—C26—H26B	109.00
P1—C1—C2	118.7 (4)	H26A—C26—H26C	110.00
P1—C1—C6	122.9 (4)	H26B—C26—H26C	109.00
C2—C1—C6	118.4 (5)	Si1—C27—H27A	110.00
C1—C2—C3	120.3 (6)	Si1—C27—H27B	110.00
C2—C3—C4	119.9 (6)	Si1—C27—H27C	109.00
C3—C4—C5	120.1 (6)	H27A—C27—H27B	109.00
C4—C5—C6	120.1 (5)	H27A—C27—H27C	109.00
C1—C6—C5	121.1 (5)	H27B—C27—H27C	109.00
P1—C7—C12	119.5 (4)	Si1—C28—H28A	110.00
P1—C7—C8	121.8 (4)	Si1—C28—H28B	109.00
C8—C7—C12	118.5 (5)	Si1—C28—H28C	109.00
C7—C8—C9	119.9 (6)	H28A—C28—H28B	109.00
C8—C9—C10	121.2 (6)	H28A—C28—H28C	110.00
C9—C10—C11	119.5 (6)	H28B—C28—H28C	109.00
C10—C11—C12	121.2 (6)	Si2—C29—H29A	110.00
C7—C12—C11	119.8 (6)	Si2—C29—H29B	109.00
P2—C13—C14	120.8 (4)	Si2—C29—H29C	109.00
P2—C13—C18	119.6 (4)	H29A—C29—H29B	109.00
C14—C13—C18	119.1 (5)	H29A—C29—H29C	110.00
C13—C14—C15	120.8 (6)	H29B—C29—H29C	109.00
C14—C15—C16	119.9 (6)	Si2—C30—H30A	109.00
C15—C16—C17	120.4 (6)	Si2—C30—H30B	109.00
C16—C17—C18	120.2 (6)	Si2—C30—H30C	109.00
C13—C18—C17	119.6 (5)	H30A—C30—H30B	109.00
P2—C19—C20	122.0 (5)	H30A—C30—H30C	110.00
P2—C19—C24	118.1 (4)	H30B—C30—H30C	109.00
C20—C19—C24	119.7 (6)	Si2—C31—H31A	109.00
C19—C20—C21	120.1 (7)	Si2—C31—H31B	109.00
C20—C21—C22	119.4 (7)	Si2—C31—H31C	109.00
C21—C22—C23	121.4 (7)	H31A—C31—H31B	110.00
C22—C23—C24	119.0 (7)	H31A—C31—H31C	109.00
C19—C24—C23	120.5 (6)	H31B—C31—H31C	109.00
P1—C25—P2	119.6 (3)	Si2—C32—H32A	110.00
Se1—C25—P1	119.7 (3)	Si2—C32—H32B	109.00
Se1—C25—P2	116.7 (3)	Si2—C32—H32C	110.00
C1—C2—H2	120.00	H32A—C32—H32B	109.00
C3—C2—H2	120.00	H32A—C32—H32C	110.00
C2—C3—H3	120.00	H32B—C32—H32C	109.00
C4—C3—H3	120.00	Si2—C33—H33A	109.00
C3—C4—H4	120.00	Si2—C33—H33B	109.00
C5—C4—H4	120.00	Si2—C33—H33C	110.00
C4—C5—H5	120.00	H33A—C33—H33B	109.00
C6—C5—H5	120.00	H33A—C33—H33C	110.00
C1—C6—H6	119.00	H33B—C33—H33C	110.00
C5—C6—H6	119.00		
			
Se1^i^—Se1—C25—P1	98.8 (3)	C13—P2—C25—P1	84.4 (4)
Se1^i^—Se1—C25—P2	−103.8 (3)	C19—P2—C25—Se1	45.3 (4)
C25—Se1—Se1^i^—C25^i^	−180.0 (3)	C19—P2—C25—P1	−157.3 (3)
C1—P1—N1—Si1	90.8 (4)	C26—Si1—N1—P1	65.2 (5)
C7—P1—N1—Si1	−25.6 (5)	C27—Si1—N1—P1	−56.9 (5)
C25—P1—N1—Si1	−148.0 (4)	C28—Si1—N1—P1	−176.3 (4)
N1—P1—C1—C2	8.8 (6)	C29—Si2—N2—P2	−174.0 (5)
N1—P1—C1—C6	−171.4 (5)	C30—Si2—N2—P2	−64.2 (6)
C7—P1—C1—C2	131.5 (5)	C31—Si2—N2—P2	57.2 (7)
C7—P1—C1—C6	−48.8 (5)	P1—C1—C2—C3	178.2 (5)
C25—P1—C1—C2	−114.0 (5)	C6—C1—C2—C3	−1.6 (10)
C25—P1—C1—C6	65.8 (5)	P1—C1—C6—C5	−178.5 (5)
N1—P1—C7—C8	90.3 (5)	C2—C1—C6—C5	1.2 (9)
N1—P1—C7—C12	−84.4 (5)	C1—C2—C3—C4	1.4 (11)
C1—P1—C7—C8	−30.7 (5)	C2—C3—C4—C5	−0.8 (11)
C1—P1—C7—C12	154.6 (5)	C3—C4—C5—C6	0.4 (10)
C25—P1—C7—C8	−145.6 (5)	C4—C5—C6—C1	−0.6 (10)
C25—P1—C7—C12	39.6 (5)	P1—C7—C8—C9	−174.4 (5)
N1—P1—C25—Se1	161.3 (3)	C12—C7—C8—C9	0.4 (9)
N1—P1—C25—P2	4.6 (4)	P1—C7—C12—C11	176.1 (5)
C1—P1—C25—Se1	−75.8 (4)	C8—C7—C12—C11	1.3 (9)
C1—P1—C25—P2	127.4 (3)	C7—C8—C9—C10	−1.7 (10)
C7—P1—C25—Se1	35.6 (4)	C8—C9—C10—C11	1.4 (10)
C7—P1—C25—P2	−121.1 (3)	C9—C10—C11—C12	0.3 (10)
C13—P2—N2—Si2	99.9 (5)	C10—C11—C12—C7	−1.6 (10)
C19—P2—N2—Si2	−15.4 (5)	P2—C13—C14—C15	−171.8 (5)
C25—P2—N2—Si2	−137.5 (4)	C18—C13—C14—C15	−0.3 (10)
N2—P2—C13—C14	−173.2 (5)	P2—C13—C18—C17	171.2 (5)
N2—P2—C13—C18	15.3 (6)	C14—C13—C18—C17	−0.4 (10)
C19—P2—C13—C14	−56.9 (6)	C13—C14—C15—C16	1.2 (10)
C19—P2—C13—C18	131.7 (5)	C14—C15—C16—C17	−1.3 (11)
C25—P2—C13—C14	63.9 (6)	C15—C16—C17—C18	0.6 (11)
C25—P2—C13—C18	−107.6 (5)	C16—C17—C18—C13	0.3 (10)
N2—P2—C19—C20	97.1 (5)	P2—C19—C20—C21	−176.3 (5)
N2—P2—C19—C24	−77.5 (5)	C24—C19—C20—C21	−1.8 (9)
C13—P2—C19—C20	−19.3 (6)	P2—C19—C24—C23	176.8 (4)
C13—P2—C19—C24	166.1 (4)	C20—C19—C24—C23	2.1 (8)
C25—P2—C19—C20	−140.3 (5)	C19—C20—C21—C22	0.7 (10)
C25—P2—C19—C24	45.1 (5)	C20—C21—C22—C23	0.2 (10)
N2—P2—C25—Se1	166.0 (3)	C21—C22—C23—C24	0.1 (9)
N2—P2—C25—P1	−36.6 (4)	C22—C23—C24—C19	−1.2 (8)
C13—P2—C25—Se1	−73.0 (4)		

Symmetry code: (i) −*x*+1, −*y*+1, −*z*.

### Hydrogen-bond geometry (Å, º)

**Table:**

*D*—H···*A*	*D*—H	H···*A*	*D*···*A*	*D*—H···*A*
N2—H2*N*···N1	0.99 (8)	1.86 (8)	2.795 (8)	156 (6)
